# Profiles of Small Non-Coding RNAs in *Schistosoma japonicum* during Development

**DOI:** 10.1371/journal.pntd.0001256

**Published:** 2011-08-02

**Authors:** Pengfei Cai, Nan Hou, Xianyu Piao, Shuai Liu, Haiying Liu, Fan Yang, Jianwei Wang, Qi Jin, Heng Wang, Qijun Chen

**Affiliations:** 1 Laboratory of Parasitology, Institute of Pathogen Biology/Institute of Medical Sciences, Chinese Academy of Medical Sciences and Peking Union Medical College, Beijing, China; 2 Key Laboratory of Zoonosis, Ministry of Education, Institute of Zoonosis, Jilin University, Changchun, China; University of Queensland, Australia

## Abstract

**Background:**

The gene regulation mechanism along the life cycle of the genus *Schistosoma* is complex. Small non-coding RNAs (sncRNAs) are essential post transcriptional gene regulation elements that affect gene expression and mRNA stability. Preliminary studies indicated that sncRNAs in schistosomal parasites are generated through different pathways, which are developmentally regulated. However, the data of sncRNAs of schistosomal parasites are still fragmental and a complete expression profile of sncRNAs during the parasite development requires a deep investigation.

**Methodology/Principal Findings:**

We employed high-throughput genome-wide transcriptome analytic techniques to explore the dynamic expression of microRNAs (miRNAs) and endogenous siRNAs (endo-siRNAs) of *Schistosoma japonicum* covering the free-living cercarial stage and all stages in the definitive host. This led us to analyze over 70 million clean reads represented both high and low abundance of the small RNA population. Patterns of differential expression of miRNAs and endo-siRNAs were observed. MiRNAs was twice more than endo-siRNAs in cercariae, but gradually decreased along with the development of the parasite. Both small RNA types were presented in equal aboudance in lung-stage schistosomula, while endo-siRNAs accumulated to 6 times more than miRNAs in adult female worms and hepatic eggs. Further, miRNAs were found mainly derived from genes located in the intergenic regions, while endo-siRNAs were mainly generated from transposable elements (TEs). The expression pattern of TE-siRNAs, as well as the pseudogene-derived siRNAs clustered in mRNAs of cytoskeletal proteins, stress proteins, enzymes related to energy metabolism also revealed distinction throughout different developmental stages. Natural antisense transcripts (NATs)-related siRNAs accounted for minor proportion of the endo-siRNAs which were dominantly expressed in cercariae.

**Conclusions/Significance:**

Our results represented a comprehensive expression profile of sncRNAs in various developmental stages of *S. japonicum* with high accuracy and coverage. The data would facilitate a deep understanding of the parasite biology and potential discovery of novel targets for the design of anti-parasite drugs.

## Author Summary

Schistosomiasis, a debilitating disease, caused by agents of the genus *Schistosoma* afflicts more than 200 million people worldwide. Schistosomes could serve as an interesting model to explore gene regulation due to its evolutional position, complex life cycle and sexual dimorphism. We previously indicated that sncRNA profile in the parasite *S. japonicum* was developmentally regulated in hepatic and adult stages. In this study, we systematically investigated mircoRNA (miRNA) and endogenous siRNA (endo-siRNA) profile in this parasite in more detailed developmental stages (cercariae, lung-stage schistosomula, separated adult worms, and liver tissue-trapped eggs) using high-throughput RNA sequencing technology. We observed that the ratio of miRNAs to endo-siRNAs was dynamically changed throughout different developmental stages of the parasite. MiRNAs were expressed dominantly in cercariae, while endo-siRNAs accumulated in adult female worms and hepatic eggs. We demonstrated that miRNAs were mostly derived from intergenic regions whereas siRNAs were mostly derived from transposable elements. We also annotated miRNAs and siRNAs with stage- and gender- biased expression. Our findings would facilitate to understand the gene regulation mechanism of this parasite and discover novel targets for anti-parasite drugs.

## Introduction

Schistosomiasis is a chronic debilitating disease that afflicts more than 200 million individuals in the tropics and sub-tropics regions [1]. The agents of this disease, parasitic flatworms of the genus *Schistosoma*, have a complex developmental life cycle characterized by a distinct parasitic phase in mammalian and molluscan hosts and a free-living phase in freshwater. There are at least seven discrete developmental stages of the parasite within the definitive (lung-stage schistosomula, juvenile, adult male and female worms, and eggs) and intermediate (sporocysts) hosts as well as the aquatic, free-swimming miracidia and cercariae, with dramatically morphological changes [2]. And they are among the few platyhelminth parasites to adopt a dioecious lifestyle and possess heteromorphic sex chromosomes, which are arrayed in 7 pairs of autosomal chromosomes and one pair of sexual chromosomes (Z, W), homozygous (ZZ) for male and heterozygous (ZW) for female [3], [4]. Previous investigations on *Schistosoma japonicum* had revealed that a complex gene regulation pattern was deployed by this genus of parasites [5] and its haploid genome, which is about 270 Mb in size, has been recently decoded as a valuable entity for identification of small regulatory RNAs of this parasite [6].

Small non-coding RNA transcripts, approximately 18–30 nucleotides in length, are critical regulators in silencing of target genes in fungi, plants, and metazoans [7]–[9]. Three major categories of sncRNAs, siRNAs, miRNAs, and Piwi-interacting RNAs (piRNAs) have been well established and extensively studied [10]. SncRNAs exert their regulatory functions in chromatin architecture modelling, post-transcriptional repression and mRNA destabilization, mobile genetic elements suppression, and virus defence, usually through guiding the RNA-induced silencing complex (RISC) to their target genes [7], [11]–[13]. In *Drosophila*, sncRNAs are generated through Dicer-dependent or independent pathways [14]. Dicer-1 generates miRNAs whereas Dicer-2 creates endo-siRNAs. Recently, it was found that the Argonaute protein family, which include the ubiquitous AGO (AGO1 and AGO2) and the germline-specific Piwi (AGO3) were devoted to different small RNA-mediated regulatory pathways [15]. AGO1 functions primarily in the miRNA-dependent pathway that silences messenger RNA, whereas AGO2 has been involved in RNAi-mediated silencing directed by exogenous and endogenous siRNAs. Further study in *Drosophila* somatic cells revealed that there were two classes of endo-siRNAs, one was generated from TEs and involved in retrotransposon repression; the other was produced in a Dicer-2-dependent manner from distinctive genomic loci, through refolding of RNA transcripts. The function of the second class of endo-siRNAs was likely to regulate mRNA stability in somatic cells [14].

Recently, several groups have endeavored to identify and characterize sncRNAs of schistosome with conventional cloning method and the deep-sequencing technique, mainly focused on juvenile and mixed adult worms, the two relatively closed developmental stages of the parasite [16]–[21]. A repertoire of miRNA transcripts unique to *S. japonicum* or those conserved to other metazoan lineages was identified. Differential expression of certain miRNAs was observed between the two developmental stages of *S. japonicum* (hepatic schistosomula and adult worms) and *S. mansoni* (7d schistosomula and adult worms), suggesting that miRNAs play a distinct role in modulating development, maturation, and reproduction of the parasite [17]–[19], [21]. Importantly, miRNA genes within one cluster could be differentially expressed, which emphasized that individual transcript might be developmentally regulated by distinct mechanisms [17], [19]. Meanwhile, a set of endo-siRNAs derived mainly from transposable elements (TEs) and the natural antisense transcripts (NATs) of *S. japonicum* has also been defined [17], [19]. Interestingly, the distinct length and 3′ end heterogeneity of endo-siRNAs derived from both TEs and NATs were also associated with the developmental differentiation of the parasite [17].

Though the knowledge regarding sncRNA biology within the juvenile and mixed adult worms of *S. japonicum* is expanding, it is indispensable to systematically profile the repertoire of sncRNAs in other stages, especially the cercariae, which is the only infectious stage to penetrate its mammalian hosts; the lung-stage schistosomula, that is viewed as the most susceptible stage for intervention [22], [23]; the tissue trapped eggs, which is the critical mediator for severe pathology in schistosomiasis, and the difference between the two sexes of adult worms. In this study, the expression profile of sncRNAs in the four critical developmental stages of *S. japonicum* was systematically investigated. The data, for the first time, provide a broader view of small non-coding RNAs in the parasite.

## Materials and Methods

### Parasites and animals

The freshly released cercariae of *S. japonicum* were harvested from parasite-infected *Oncomelania hupensis* purchased from Jiangxi Institute of Parasitic Diseases, Nanchang, China. The lung-stage schistosomula (3 days post infection) were isolated from lung tissues of infected Kunming strain mice as previously described [24]. Adult worms were obtained by hepatic-portal perfusion of New Zealand White rabbits or BALB/c mice 7-weeks post infection. Male and female worms were manually separated with the aid of a light microscope. Liver tissues deposited with schistosome eggs were obtained from BALB/c mice at day 30 and 45 post infection, respectively. All procedures performed on animals within this study were conducted following animal husbandry guidelines of the Chinese Academy of Medical Sciences and with permission from the Experimental Animal Committee. All animal work have been conducted according to Chinese and international guidelines.

### Total RNAs isolation

Total RNAs of *S. japonicum* at different developmental stages (cercariae, lung-stage schistosomula, adult male and female worms perfused from infected rabbits) and liver total RNAs of BALB/c mice 30d and 45d post infection were extracted using Trizol reagent (Invitrogen, CA, USA). RNA quantification and quality were evaluated by Nanodrop ND-1000 spectrophotometer (Nanodrop Technologies, Wilmington, DE) and Agilent 2100 Bioanalyzer (Agilent Technologies, Palo Alto, CA).

### Small RNA libraries construction and sequencing

Construction of small RNA libraries was carried out as described previously. Briefly, RNAs between 15–30 nucleotides (nt) were excised from a 15% TBE urea polyacrylamide gel electrophoresis (PAGE). RNA samples were purified and ligated to Illumina's proprietary 5′ and 3′ adaptors, and further converted into single-stranded cDNA with Superscript II reverse transcriptase (Invitrogen, CA, USA) and Illumina's small RNA RT-Primer. The cDNA was amplified with high fidelity Phusion DNA polymerase (Finnzymes Oy, Finland) in 18 PCR cycles using Illumina's small RNA primer set. The purified PCR products were sequenced by an Illumina Genome Analyzer at the BGI (Beijing Genomics Institute, Shenzhen, China).

### Mapping sequence reads to the reference genome

Raw datasets produced by deep sequencing from the libraries (cercariae, lung-stage schistosomula, adult male and female worms, and infected liver tissues) were pooled. Clean reads were obtained after removing of the low quality reads, adaptor null reads, insert null reads, 5′ adaptor contaminants, and reads with ployA tail. Adapter sequences were then trimmed from both ends of clean reads. All identical sequences were counted and merged as unique sequences, herein referred to as sequence tags. The unique reads along with associated read counts were mapped to the *S. japonicum* genome sequences (http://lifecenter.sgst.cn/schistosoma/cn/schdownload.do) using the program SOAP [25]. As for the liver libraries, the unique reads were mapped to the genome of mouse (http://hgdownload.cse.ucsc.edu/downloads.html#mouse) with SOAP, and those perfectly matched ones were removed prior to mapping to the *S. japonicum* genome.

### Bioinformatic analysis of *S. japonicum* small RNAs

Briefly, the perfectly matched reads were first BLAST-searched against the 78 known mature miRNAs of *S. japonicum* deposited in Sanger miRBase [26], [27] (Release 15) using the program Patscan [28]. The remains were then BLAST-searched against Metazoa other than *S. japonicum* miRNAs allowing two mismatches to identify homologs of known Metazoa miRNAs. These homologs, as well as non-conserved reads (with rRNA, tRNA, snoRNA and high repetitive reads being filtered out [29]) were considered as potential miRNAs. To avoid repeated prediction and reduce the calculation redundancy, we then searched against the genome of *S. japonicum* and combined candidate unique reads located in close proximity in the reference genome with less than 150 bp and we called the joint genomic fragment as a block. For each block, 150 nt upstream and 150 nt downstream sequence were extracted for secondary structure analysis. We used software Einverted of Emboss [30] to find the inverted repeats (step loops or hairpin structure), with the parameters threshold  = 30, match score  = 3, mismatch score  = 3, gap penalty  = 6, and maximum repeat length  = 240 as described [31]. Each inverted repeat was extended 10 nt on each side, the secondary structure of the inverted repeat was folded using RNAfold [32] and evaluated by mirCheck using default parameters [31]. MiRNA candidates passed mirCheck were Blast-searched against Metazoa miRNAs except those of *S. japonicum* using the program Patscan again and labeled with conserved and non-conserved (novel) miRNAs, respectively. The novel unique reads that sequenced less than 2 times were removed. Finally, miRNA precursors that passed MirCheck were inspected manually in order to remove the false prediction. We employed IDEG6 to identify miRNAs showing statistically significant difference in relative abundance (as reflected by TPM value) between any two small RNA libraries [33]. The general Chi2×2 test was applied to determine whether one particular miRNA was significantly differentially expressed between any two samples (*P* value < = 0.01). Hierarchical clustering of the known *S. japonicum* miRNAs was constructed based on the transformed data of log_10_ of TPM value.

The transposable elements in the *S. japonicum* genome were predicted by using REPET (http://urgi.versailles.inra.fr/index.php/urgi/Tools/REPET). TE-derived siRNAs were identified as previously described [17]. Figures were constructed to reflect the relative abundance of sense and antisense of TE-derived siRNAs during the parasite development. Briefly, the expression value of each base on TE was the sum of the expression of siRNAs that mapped to the position. After a proper bin (20–50 bases) was selected based on the length of TE sequences, the average expression value was calculated for each bin, and the expression level for four stages was marked by different colors. The natural antisense transcripts of *S. japonicum* were annotated and NAT-derived siRNAs were confirmed as described [17]. The small RNAs that failed to pass mirCheck were aligned to *S. japonicum* predicted mRNA sequences of SGST (http://lifecenter.sgst.cn/schistosoma/cn/schdownload.do) using the program SOAP, and perfectly matched reads were retained. Then a Perl script was wrote to scan the predicted mRNAs, if the region continuous covered by small RNAs is longer than 100 bp, the region was deemed as a “siRNA-Cluster”.

### Quantitative RT-PCR analysis of sex-biased miRNAs

Stem-loop qRT-PCR was performed to quantitate the sex-biased expressed miRNAs [20], [34]. Stem-loop RT primers were designed to reverse-transcribe target miRNAs into cDNAs using total RNAs isolated from male and female adult worms, respectively (from the same smaples used for Solexa sequecing). The 20 µl reaction system contained 1 µg of total RNA, 50 nM of each individual stem-loop RT primer, 1×RT buffer, 0.5 mM dNTPs (Takara), 0.01 M DTT (Invitrogen), 0.25 µl Superscript III reverse transcriptase (200 U/µl, Invitrogen, CA, USA), and 0.1 µl RNaseOUT inhibitor (40 U/µl, Invitrogen). cDNA was synthesized by incubation for 30 min at 16°C, 30 min at 42°C, 15 min at 70°C. Real-time quantification was carried out using an Applied Biosystems StepOne Plus system. PCR reactions were set up by combining 0.5 µM miRNA-specific forward primer, 0.5 µM common reverse primer, 2 µl of RT product (1∶1 dilution), 10 µl of Power SYBR Green PCR Master Mix (ABI, CA, USA), and adjusted to a final volume of 20 µl with DEPC-treated water in triplicates. For endogenous control, 1 µg of male or female total RNA was converted to cDNA with oligo(dT). The forward primer: 5′-CCTTCATGGTAGACAACGAAGCT-3′ and reverse primer: 5′-TGTAGGTTGGACGCTCTATGTCC-3′, were used to amplify the α-tubulin gene as an endogenous control. The reaction conditions were as follows: 95°C for 5 min, followed by 40 cycles of 95°C for 5 sec and 60°C for 30 sec. The quantification of each miRNA relative to α-tubulin mRNA was calculated using the equation: N = 2^−ΔCt^, ΔCt  =  Ct_miRNA_ - Ct_α-tubulin_ [35]. All primers used are listed in Table S1.

### Northern blot

5′-DIG-labeled miRCURY LNA probes were ordered from Exiqon (Vedbaek, Denmark) (Http://www.exiqon.com). Northern blot analysis was performed mianly by a method described in a previous study [36]. Total RNAs were isolated from male adult female adult worms perfused from BALB/c mice 7-weeks post infection. 10 µg total RNA of each smaple was resolved by 15% denaturing (7 M urea) PAGE and were blotted by capillary transfer to neutral Nylon Membranes (Hybond-NX, GE) with 20×SSC. RNAs were further cross-linked to the membrane by EDC (1-ethyl-3-(3-dimethylaminopropyl) carbodiimide) method [37]. Blots were pre-hybridized by incubation with DIG Easy Granule (Roche) at 37°C for 3 h. And hybridization were carried out in the same buffer containing 1 nM DIG-labeled LNA probe at temperature recommended by manufacturer (RNA Tm - 30°C) overnight. Blots were washed twice in a low stringently buffer (2×SSC, 0.1% w/v SDS), and four times in a high stringently buffer (0.1×SSC, 0.1% w/v SDS), for 30 min each, both at hybridization temperature. The membrane was rinsed in washing buffer, and incubated in blocking solution at room temperature for at least 2 h (DIG washing buffer and blocking solution Set, Roche). Subsequently, blots were incubated with a 10,000-fold dilution of anti-DIG-AP (Roche) in blocking solution at room temperature for 30 min, washed 5 times for 15 min each in washing buffer. After rinsing in detection buffer for 5 min, the blots were detected using CDP-star chemiluminescent substrate for alkaline phosphatase (Roche). Blots were stripped by boiling for 1 min at 100°C in 10 mM Tris-HCl, pH 8.0, 5 mM EDTA, and 0.1% SDS and probed up to three times.

## Results and Discussion

### Small RNA distribution in libraries from various developmental stages

Six small RNA libraries were generated by high-throughput RNA sequencing (see Materials and Methods and Table S2). Four libraries, SjC, SjL, SjM, and SjF, were constructed from sequences that were directly derived from the cercariae, lung schistosomula, adult male, and female worms, respectively. The two remaining libraries, SjE30 and SjE45, were egg libraries derived from the hepatic tissues of BALB/c mice 30 and 45 days post-infection, respectively. The reads that aligned to the mouse genome were filtered before they were mapped to the genome of *S. japonicum*. In total, 65,630,916 clean reads were obtained from libraries SjC, SjL, SjM, and SjF, which were merged into 6,989,949 unique tags, thus resulted in an average redundancy as high as 89.3 (Redundancy = 100-(Total Unique Tags/Total Clean Reads ×100)). Among these unique tags, 1,593,604 (22.80%) could be aligned to the genome of *S. japonicum* (Table S3). The match rate was varied among different libraries, from the lowest of 20.46% (SjM) to the highest of 31.95% (SjF), this phenomenon may related with the change of ratio of different small RNAs during development and/or between sexes. The low match ratio to the genome may be caused by either genome variation of different parasite isolates or due to less sequence information of the intergenic regions where most of the miRNAs were generated. The phenomenon was also observed in similar studies by others, and several explanations have been offered [18]. Regarding the egg libraries, 15,774 and 20,800 unique tags from libraries SjE30 and SjE45, respectively, mapped to the *S. japonicum* genome (Table S4). These datasets contain roughly an order of magnitude more sequence than previous similar studies.

**Figure 1:**
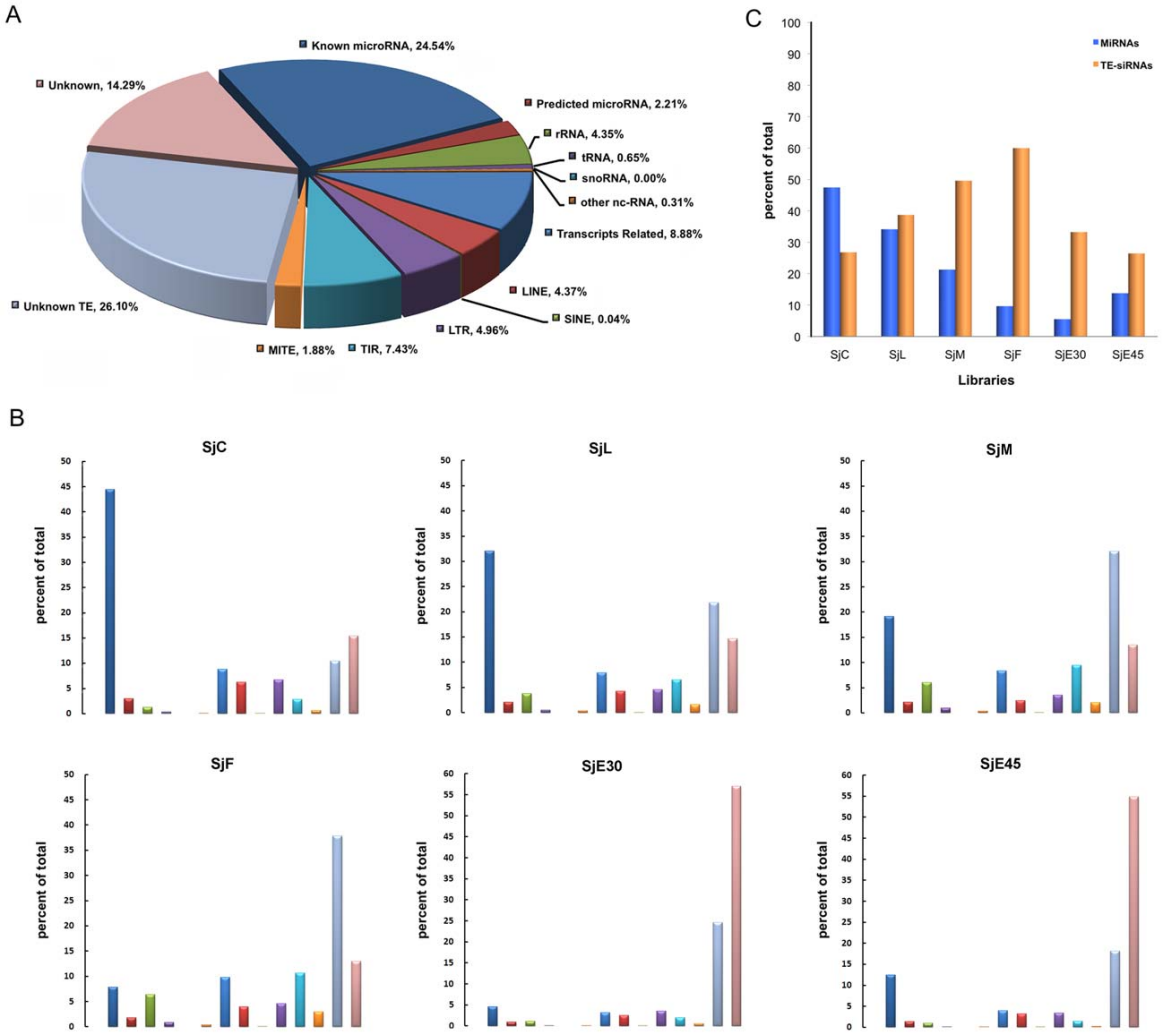
Classification and percentage of *S. japonicum* sncRNA. **A**. Classification and percentage of *S. japonicum* sncRNA using mixed small RNA data from all stages sequenced. MiRNAs took up more than 25% of total small RNAs. TE-siRNAs were mainly derived from LTR, LINE, TIR, and MITE, and accounted for approximately 45% of total small RNAs (including those from unknown TEs). **B**. Classification and percentage of *S. japonicum* sncRNA from different developmental stages. **C**. The percentage of miRNAs and TE-siRNAs during different developmental stages. The ratio of siRNA to miRNA was gradually increased and accumulated to the top in female worms and early deposited eggs. Note that the color scheme used in section B was the same as section A.

The short ncRNA transcripts were categorized according to features related to primary and secondary structure (Figure 1 and Table S5). The majority of the ncRNA transcripts were miRNAs and TE-derived endo-siRNAs, accounting for 26.75% and 44.77%, respectively, of the total sncRNA pool (Figure 1A). Only 2.21% of the miRNAs identified in our libraries were novel, indicating that most miRNAs have been recovered from the genome. Long terminal retrotransposons (LTR) and un-annotated transposons were predominant in the set of endo-siRNAs. Interestingly, the sets of miRNAs and endo-siRNAs displayed stage- or sex-related variation in expression (Figure 1B and C). The percentage of miRNA was approximately double than that of the TE-derived endo-siRNA set in the cercariae library; the amount of miRNAs and endo-siRNAs was almost equal in lung-stage schistosomula, while endo-siRNAs were dominant in the adult worms and eggs, especially in female worms and early deposited eggs (6 times more than that of miRNAs). A class of endo-siRNAs derived from unclassified transposons was dominantly expressed in the male and female parasite compared to other stages (Figure 1B). The clear pattern of preferential expression of the genes encoding the two classes of small RNAs suggests that they play stage-specific regulatory functions. Before invasion into a mammalian host, the parasite is likely to mainly utilize miRNA pathways to regulate gene expression, while endo-siRNA mediated regulation is suppressed. The high percentage of TE-derived endo-siRNAs in females and early deposited eggs suggests that siRNAs are more functional at these developmental stages. Earlier studies in *D. melanogaster* and mouse oocytes demonstrated that endo-siRNAs were critical elements for maintaining genomic stability through suppression of TE activity [38]–[40]. *S. japonicum* possesses a faster reproductive rate than flies or mice, and thousands of eggs are released by one female adult worm each day. Efficient suppression of TE activity is likely a prerequisite for continuity of parasite development and transmission, a possible explanation for why TE-derived endo-siRNAs were dominantly found in late-stage parasites.

### miRNAs identified in different stages of *S. japonicum*

When the sequences of the small RNAs containing classical miRNA structure were aligned to the Sanger miRBase (Release 15), 77 sequences homologous to known or well-characterized miRNAs were identified. We found 74, 71, 69, 70, 18, and 25 such sequences in libraries SjC, SjL, SjM, SjF, SjE30, and SjE45, respectively. Only one miRNA, the previously reported sja-miR-8-5p [19], was not detected in this study (Table S6). Among the set of 77 known miRNAs, approximately 20 miRNAs were conserved homologues of sequences from the planarian *Schmidtea mediterranea*, the genus most closely related to *Schistosoma*, in previous investigations [17], [19], [20], [41]–[43], indicated that phylum Platyhelminthes contains common miRNAs that carry out similar biological function. The maximum read number of a single miRNA was 1,044,358 (library SjC, sja-miR-1; Table S7), illustrating the sequencing depth of our investigation. The range of read numbers was from the single-digits to millions, highlighting the sequencing capacity of next-generation sequencing technology and suggesting that expression variation of these miRNAs does indeed exist. This observation most likely reflects functional differentiation among the miRNAs.

Apart from the known miRNAs, 193 hairpins containing 45 conserved mature miRNAs derived from 19 families were predicted in our sequence libraries. These miRNAs along with their expression level (reflected by transcripts per million, TPM) during development were shown in Table S8. Additionally, we identified 199 novel miRNAs with various expression levels and stage specificities (Table S9). In contrast with the common or evolutionarily conserved miRNAs, most novel miRNAs identified in this study possessed low read numbers, with the exceptions of sequences sja-novel-23-5p and sja-novel-48-3p, which was mainly expressed in female adult worms and cercariae, respectively.

**Figure 2:**
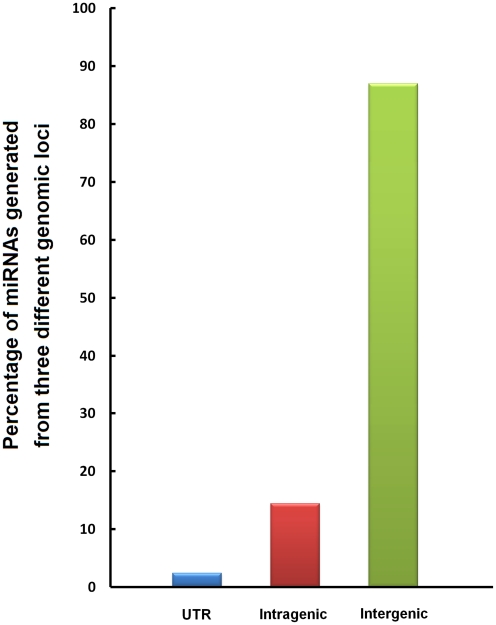
Percentage of miRNAs generated from three different genomic loci in *S. japonicum*. MiRNA transcripts identified in *S. japonicum* genome were derived from up- and down-stream UTR, intragenic and intergenic regions. Proportion of the miRNAs from the three regions was 2.4%, 14.4%, and 87.2%, respectively.

Previous investigations of miRNA biogenesis revealed that miRNA genes are located either in intergenic regions [44] that are controlled by their own miRNA promoters and regulatory units [45], or in introns, non-protein coding genes, or exons, and thus they are likely to be regulated in concert with host genes [46]. In an earlier study, we found that many *S. japonicum* miRNA genes were clustered together, and that genes within the same cluster may be regulated independently [17]. In the present study, we mapped all identified miRNA sequences to the *S. japonicum* genome and found that miRNAs were generated from 5′ or 3′ UTRs, intragenic, and intergenic regions in the genome; however, a majority of sequences (87.2% of the total miRNAs identified) were transcripts derived from genes located in intergenic loci (Figure 2). Thus, compared to *Caenorhabditis elegans*, *S. japonicum* has evolved more sophisticated control mechanisms for regulation of miRNA expression, possibly explaining the complicated nature of the transcription profiles of individual miRNAs in various developmental stages of the parasite.

### Differential expression of miRNAs during parasite development

**Figure 3:**
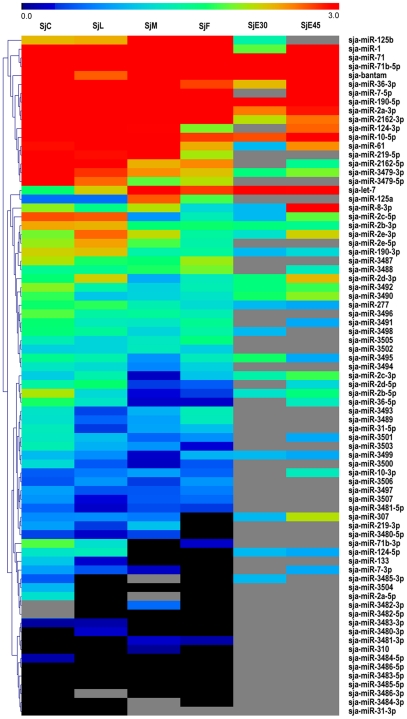
Hierarchical clustering of the known miRNAs during different developmental stages using Pearson correlation. Heatmap was constructed based on the log_10_ of TPM value of miRNAs. Black indicated the expression value of the miRNA was 0 after normalization. Grey indicated that the miRNA was not detected in that library. TPM value for each tag was calculated by the sum of total clean reads which were within±2 bp variations of the mature miRNAs on the precursor.

Although the relative expression level of a particular miRNA has been proposed to be represented by the number of sequence reads, other investigations have argued that neither read counts nor northern blot signal accurately reflect actual abundance or expression level [19], [47], [48]. Here, the expression levels of each unique tag in cercariae, lung-stage schistosomula, separated adult worms and eggs libraries were normalized to TPM as previously described [18], [49], [50]. Thus, the read abundance should basically reflect the expression level of the tags in the parasites. The scale of the relative miRNA abundance during the various developmental stages appears in Figure 3. Of 77 known miRNAs, 28 miRNAs exhibited high expression levels in one or more developmental stages. The expression levels of the novel miRNAs identified in this study were generally low (Table S9). However, four miRNAs were with relatively higher expression level in one particular stage, as sja-Novel-23-3p and sja-Novel-23-5p were dominantly expressed in the female parasite, while sja-Novel-48-3p and sja-Novel-74-3p were substantially expressed in cercarial stage.

Like *C. elegans*, schistosomal parasites need to complete a series of biological and physiological activities, including protease secretion, tail detachment, glycocalyx shedding, and tegument transformation before developing to the schistosomula stage [51], [52]. A particular gene repertoire of *S. mansoni* parasites was previously shown to be up-regulated during the transition from schistosomula to adult worms [22]. Here, we observed that the expression of a set of miRNAs including sja-bantam, sja-miR-1, sja-miR-124-3p, sja-miR-2a-3p, sja-miR-3492, and sja-miR-36-3p was substantially down-regulated in lung-stage schistosomula compared to cercariae (Table S6), suggesting that the target mRNAs of these miRNAs may encode proteins fulfilling important functions at this stage.

### Sex-biased miRNA expression

**Figure 4:**
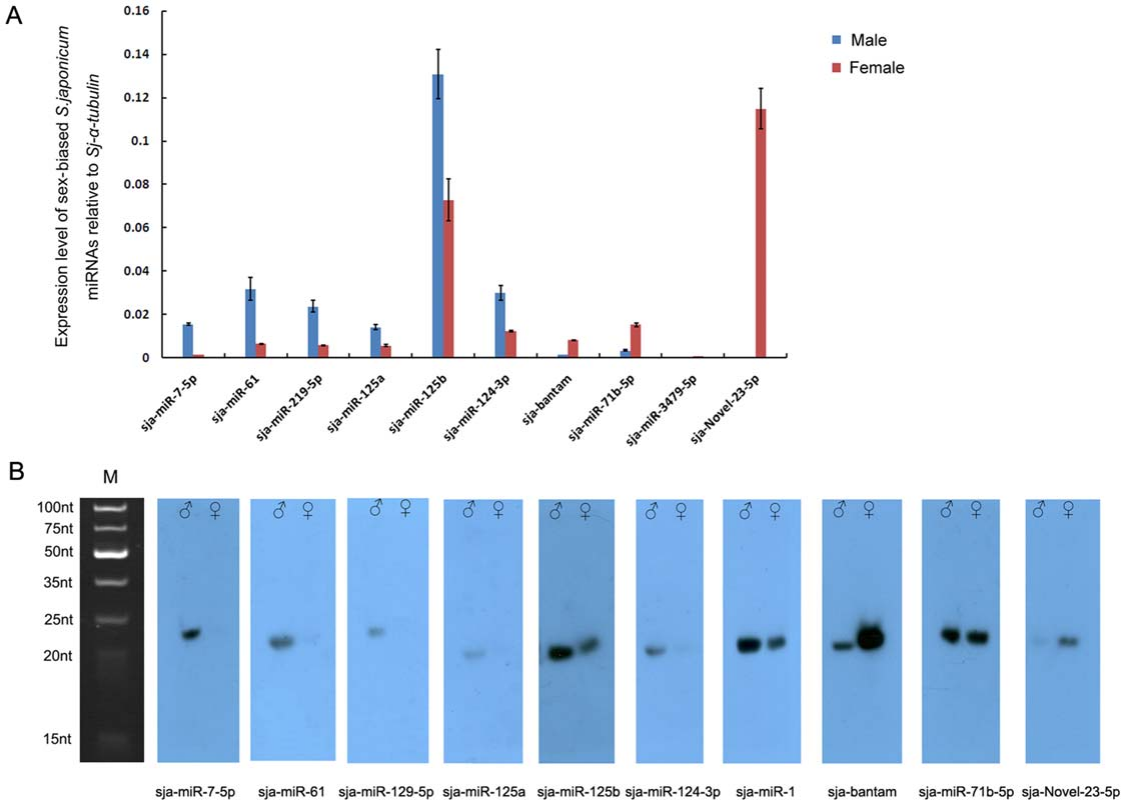
Validation of sex-biased miRNAs by quantitative RT-PCR and Northern blot. **A**. The relative abundance of 9 known miRNAs and one novel miRNA investigated by quantitative RT-PCR. Six miRNAs (from sja-miR-7-5p to sja-miR-124-3p) showed higher expression level in male parasites, while the remaining four were dominantly expressed in the female parasites. **B**. Northern blot analysis of ten sex-biased miRNAs using RNAs isolated from male and female adult worms perfused from BALB/c mice. M, ultra low range DNA ladder denatured in RNA loading Dye solution as the total RNA done. ♂: male adult worms; ♀: female adult worms.

We further explored the differential expression of miRNA genes between male and female adult worms. The expression of a set of miRNAs, sja-miR-7-5p, sja-miR-61, sja-miR-219-5p, sja-miR-125a, sja-miR-125b, sja-miR-124-3p and sja-miR-1 were dominant in male worms, while sja-bantam, sja-miR-71b-5p, sja-miR-3479-5p, and sja-Novel-23-5p were predominantly found in the female parasites (Table S6 and S9). The expression of these sex-biased miRNAs was validated by stem-loop RT-PCR (Figure 4A). The expression level of sja-miR-1 was relatively high in male adult worms (1.098±0.228) and female adult worms (0.358±0.021) when compared to other miRNAs, and was not shown in Figure 4A. The correlation between the TPM values and qPCR was investigated by a method described in a previous study (R = 0.882, Spearman's Rho, *p*<0.0001, n = 11) [53]. However, among individual miRNAs, the qPCR results did not exactly reflect the TPM values of the maximally expressed miRNAs, probably due to the existence of extensive miRNA isomiRs, or asymmetrical amplification during library construction. We further validated the expression differences of ten sex-biased miRNAs by northern blot analysis using the total RNA extracted from adult male and female worms isolated from BALB/c mice 7-weeks post infection (Figure 4B). The differential expression pattern of these miRNAs except sja-miR-71b-5p between male and female worms was quite consistent with the TPM values of high-throughput sequencing and the qRT-PCR results. The phenomenon was also observed in a recent study which noted that several miRNAs were expressed at similar levels in protoscoleces of G1 and G7 genotypes *Echinococcus granulosus*, which parasitized in different hosts [54]. Thus, these data indicated that host factors may have little impact on the expression profile or level of sncRNAs.

Although the function of these miRNAs remains to be elucidated, the significant differential expression between male and female adult worms indicated that they may participate in regulation of sexual differentiation and maintenance, pairing and reproduction of the parasite. Moreover, miRNAs may cooperate with other small RNAs (such as endo-siRNAs) and transcription factors to form a comprehensive network to regulate growth, development, differentiation, and reproduction for adaptation to a variety of environments [19]. Further studies on these miRNAs may contribute to better understanding of the developmental mechanism of sexual dimorphism in this parasite.

### TE-derived endo-siRNAs

Recent observations of endo-siRNAs in *D. melanogaster*, mice, and schistosome have added more complexity to our knowledge of small RNA-mediated regulatory pathways [14], [17], [38]–[40], [55]–[58]. So far, endo-siRNAs have been found to be mainly derived from TEs, complementary annealed NATs, and the long “fold-back” transcripts known as hairpin RNAs [59]. We previously found that the TE-derived siRNAs in *S. japonicum* were more predominant than other endo-siRNAs, including NAT-derived siRNAs [17]. Here, we systematically analyzed the expression levels of sense and antisense endo-siRNAs that derived from various TEs in cercariae, lung-stages schistosomula, male and female adult worms (Table S10–14). The read numbers of endo-siRNAs in egg libraries were much lower than the other libraries, leading us to exclude the egg libraries from further analysis.

**Figure 5:**
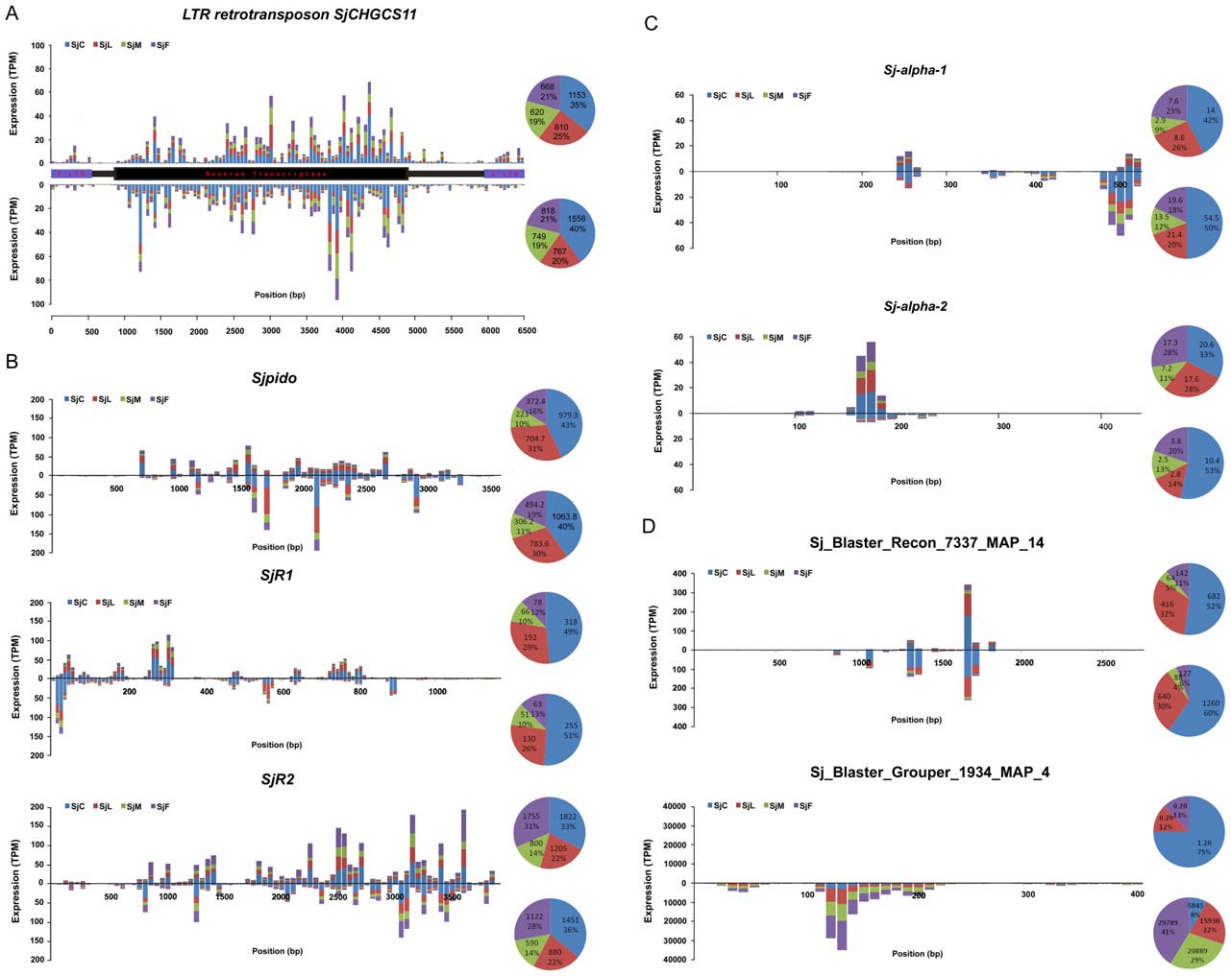
The abundance of sense and antisense siRNAs that mapped to the *S. japonicum* TEs. The TPM of the siRNA sequences generated from TEs during different developmental stages was presented with bars in different colors. **A**. Endo-siRNAs mapped to the LTR retrotransposon *SjCHGCS11*. The amount of siRNAs generated from the sense and antisense strands were similar but with different stage preferences. **B**. Endo-siRNAs mapped to TE of LINE type, *Sjpido* (upper), *SjR1* (middle), *SjR2* (lower). Compared to those from LTR, siRNAs from these TEs were transcribed from more concentrated region in the genes. **C**. Endo-siRNAs mapped to TE of SINE Type, *Sj-alpha-1* (upper), *Sj-alpha-2* (lower). **D**. Endo-siRNAs mapped to TE of TIR type (Sj_Blaster_Recon_7337_MAP_14) and MITE type (Sj_Blaster_Grouper_1934_MAP_4). The sum and percentage of TPM of sense and antisense siRNAs from each stage were displayed in Pie Chart.

We observed that LINE, TIR, and LTR transposon classes were the main sources of endo-siRNAs, while the endo-siRNAs derived from other TEs were much less abundant (Figure 5). Further, endo-siRNAs mapped to the top (sense siRNA) and bottom (antisense siRNA) strands of LTR and non-LTR TEs. The expression patterns of LTR-derived sense and antisense siRNAs were relatively symmetrical, though there were obvious stage and sex specificities in expression loci (Figure 5A, B, and C). Reads mapped to the *S. japonicum* LTR retrotransposon *SjCHGCS11* [6] were annotated as SACI-7_2p in our analysis (Figure 5A). Both sense and antisense siRNAs were concentrated in the coding region of reverse transcriptase in a manner similar to that observed in *D. melanogaster* somatic cells [38].

*Sjpido*, *SjR1*, and *SjR2* are three classes of non-LTR retrotransposons that make up 5% of the *S. japonicum* genome; siRNAs generated from these elements mainly mapped to certain sequence regions (Figure 5B), contrary to our observations of LTR retrotransposons. The expression levels of siRNAs derived from *SjR1* were much higher in cercariae than in male and female adult worms, indicating that these siRNAs are more functional in the earlier developmental stage (Figure 5B). *Sj-alpha-1* derived siRNAs were predominantly generated from the antisense strand, while *Sj-alpha-2* derived siRNAs were generated from the sense strand; however, both types of siRNAs had low expression levels (Figure 5C). In cercariae and lung-stage schistosomula, the TIR (Sj_Blaster_Recon_7337_MAP_14 annotated as SmTRC1_1p in the genome) derived siRNAs were highly expressed, while the MITE (Sj_Blaster_Grouper_1934_MAP_4) derived siRNAs were mainly expressed in the adult worms, and predominantly corresponded to the antisense strand (Figure 5D). Thus, the TE-derived endo-siRNAs of *S. japonicum* were more diverse than those found in *D. melanogaster*. Although the origin of the antisense siRNAs is not known (*cis*- or *trans*-transcription), their abundance suggests that they are stable and likely participate in regulatory pathways.

Previous studies of mouse oocytes revealed that antisense transcripts from pseudogenes formed double-strand RNAs with their functional counterparts, the sources of the endo-siRNAs, and the sense siRNAs were predominant in the endo-siRNA [55]. It has been proposed that the “passenger strand” of an siRNA is unstable due to the thermodynamic asymmetry of the two strands [60]. However, this hypothesis cannot explain our identification of many reads corresponding to the antisense siRNAs; in some cases, only the antisense strands were identified. Further dissection of the function of the two endo-siRNA classes would be an essential step toward understanding the network of gene regulation during the parasite development.

### *Trans*-NATs were the predominant sources of NAT-derived siRNAs

NAT-derived siRNAs are a second source of endo-siRNAs; these endo-siRNAs are further classified as *cis*-NAT- or *trans*-NAT-derived endo-RNAs [56], [61], [62]. *Cis*-NAT-derived endo-siRNAs are generated from transcripts from the same gene locu, while *trans*-NAT-derived endo-siRNAs come from NAT transcripts of distinct loci. We detected potential NAT pairs by aligning the predicted mRNA sequences to each other. Only one *cis*-NAT pair and 1772 *trans*-NAT pairs were identified *in silico* using data from SGST. Our sequencing results were remarkably similar to the *in silico* prediction; one *cis*-NAT pair and 225 *trans*-NAT pairs were detected (Table S15), indicating that bi-directional transcription was much less prevalent in schistosomal parasites and transcripts from duplicated genes are more common. Thus, *trans*-NAT-derived endo-siRNAs are likely the main sources of NAT-derived siRNA in *S. japonicum*, a scenario that differs from other organisms [63]. However, we cannot rule out the possibility that most of the *cis*-NAT pairs may be undetectable given the lack of information about the non-protein-coding regions of the *S. japonicum* genome. The identification of long non-coding RNAs in *S. japonicum* is still underway, and may provide an important source of NAT-derived siRNAs [64].

A previous study of *D. melanogaster* somatic cells demonstrated that endo-siRNAs mapped to protein-coding mRNAs rather than to transcripts of transposons that regulate mRNA expression [38]. Here, we also mapped the endo-siRNAs to the predicted mRNA sequences of *S. japonicum*, and found that nearly half of the siRNA-related transcripts clustered within predicted mRNAs. These mRNAs mainly encoded proteins from four categories: 1) proteins similar to pol polyprotein and endonuclease-reverse transcriptase; 2) cytoskeletal proteins such as myosin, actin, and tropomyosin; 3) enzymes or transporters such as COX1, COX2B, superoxide dismutase 1, glyceraldehyde 3-phosphate dehydrogenase, lactate dehydrogenase A, ATP-dependent RNA helicase, cation-transporting ATPase, H^+^-transporting ATPase, and cathepsin B and L; 4) stress proteins including heat shock protein, cold shock protein, and stress-induced phosphoprotein 1 (Table S16). We were unable to distinguish whether siRNAs clustered in pol polyprotein and endonuclease-reverse transcriptase transcripts were derived from retrotransposons or NATs. We speculated that some of the siRNAs in the other three categories were derived from trans-NATs formed by transcripts of pseudogenes and their parental genes, as suggested recently [65]; for example, the pseudogenes of *hsp70* and cathepsin B exist in schistosome genomes [66], [67]. Furthermore, the pseudogenes of actin, COX, GAPDH, FABP, and histone are common in mammalian genomes. Pseudogene-derived endo-siRNAs were previously detected in mouse oocytes, with two transcripts, *Hsp90ab1* (heat shock protein 90 kDa alpha, class B member 1) and *Dynll1* (dynein, light chain), possessing features similar to our findings [55]. Thus, unlike the silencing of selfish genetic elements by TE-related siRNAs, *trans*-NAT-derived endo-siRNAs mainly regulate the expression of mRNAs coding for a diverse set of proteins.

Our current study generated comprehensive profiles of endogenous small RNAs (miRNAs and endo-siRNAs) during the four crucial developmental stages of *S. japonicum*. Reverse expression patterns of miRNAs and endo-siRNAs during the parasite development and differentiation process were observed. Two classes of endo-siRNAs derived from TEs and *trans*-NATs were identified, and the LTR retrotransposon derived siRNAs were more abundant than siRNAs from non-LTR TEs. There are likely two layers of regulatory function employed by the parasite; the antisense siRNAs directly affect the stability of mRNA transcripts, while the sense siRNAs may function indirectly by affecting the amount of antisense transcripts. Thus, the small RNA-mediated network in schistosomal parasites is more complex than networks reported in other organisms.

## Supporting Information

Table S1 **Sequences of the primers used for stem-loop RT-PCR.**
(XLS)

Table S2 **General information of six small RNA libraries, including data quality.**
(XLS)

Table S3 **Data statistics for the four libraries (SjC, SjL, SjM, and SjF).**
(XLS)

Table S4 **Total data summary of the six libraries.**
(XLS)

Table S5 **Small RNA classification.**
(XLS)

Table S6 **Normalized expression level of the known miRNAs during development.**
(XLS)

Table S7 **Reads number of the known miRNAs.**
(XLS)

Table S8 **Reads number and expression level of the conserved miRNAs.**
(XLS)

Table S9 **Reads number and expression level of the novel miRNAs.**
(XLS)

Table S10 **Expression level of siRNAs derived from LTR retrotransposon in cercariae, lung-stage schistosomula, male and female adult worms.**
(XLS)

Table S11 **Expression level of siRNAs derived from LINE in cercariae, lung-stage schistosomula, male and female adult worms.**
(XLS)

Table S12 **Expression level of siRNAs derived from SINE in cercariae, lung-stage schistosomula, male and female adult worms.**
(XLS)

Table S13 **Expression level of siRNAs derived from TIR in cercariae, lung-stage schistosomula, male and female adult worms.**
(XLS)

Table S14 **Expression level of siRNAs derived from MITE in cercariae, lung-stage schistosomula, male and female adult worms.**
(XLS)

Table S15 **Reads number of NAT-derived siRNAs sequenced from cercariae, lung-stage schistosomula, male and female adult worm libraries.**
(XLS)

Table S16 **SiRNAs clustered within** ***S. japonicum*** **predicted mRNAs.**
(XLS)
